# Supplementary material for: Controlled Molecular Arrangement of Cinnamic Acid in Layered Double Hydroxide through pi-pi Interaction for Controlled Release
Source: Int J Mol Sci. 2024 Apr 19;25(8):4506. doi: 10.3390/ijms25084506 (PMC11050039; doi:10.3390/ijms25084506)
Supplement: Supplementary file 1 [file ijms-25-04506-s001.zip › ijms-2938143-supplementary.pdf]

Calculation of demanded area per unit charge in an LDH layer

As shown in the Fig S1., the red hexagon contains two Zn^{2+} ions and one Al^{3+} ion, representing a unit of $Zn_2Al(OH)_6^+$ of an LDH layer (Zn/Al ratio of 2/1). Taking into account the cell parameter a=0.305 nm, the area of the hexagon, which contains one positive charge, is approximately 0.25 nm^2. The demanded area for ZnAl-LDH with another Zn/Al ratio could also be calculated based on this schematic diagram. The demanded areas of Zn/Al=4/1 and 7/1 are 5/3 and 8/3 times larger than that of Zn/Al=2/1, giving rise to the area value of 0.42 nm^2 and 0.67 nm^2. Therefore, the three hybrids, CL1, CL2, and CL3, have demanded area per unit charge of 0.25, 0.42, and 0.67 nm^2, respectively.

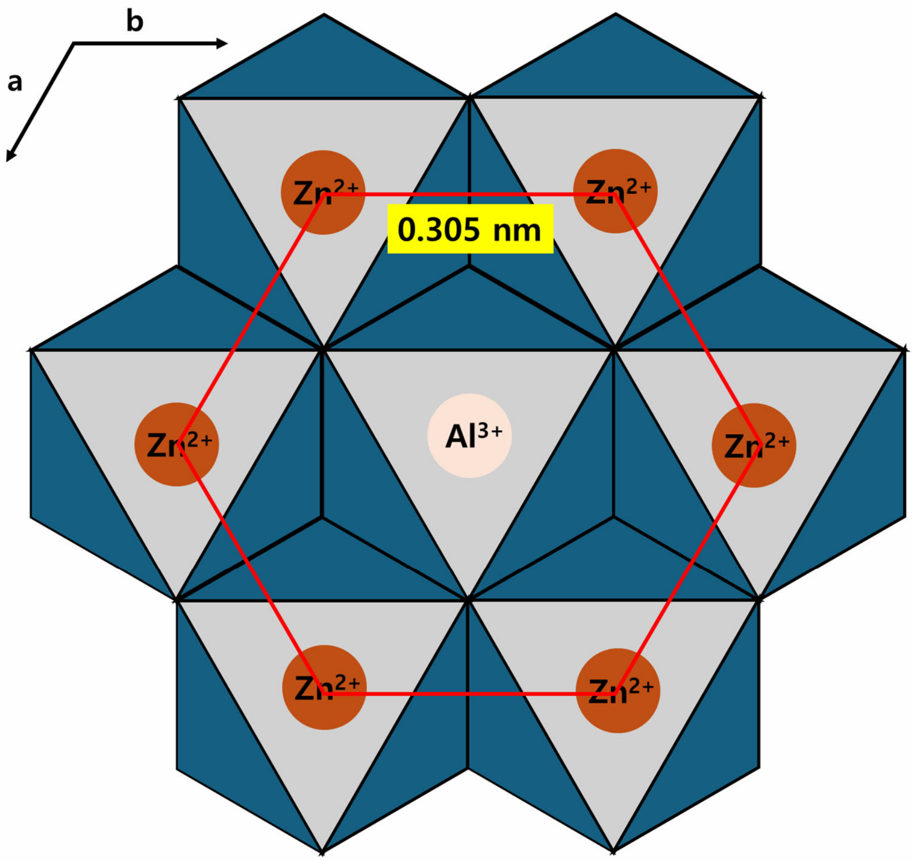

Figure. S1. Schematic diagram to calculate the demanded area for unit positive charge in ZnAl-LDH with Zn/Al ratio 2/1.

Calculation of the number of CA or CAD molecules along an LDH layer

Taking into account the molecular dimension and the intermolecular distance obtained from Monte Carlo simulation, we hypothesized that a cinnamic acid or cinnamaldehyde molecule requires cross-sectional area with diameter 0.7 nm. And the lateral dimension of one hybrid layer was approximated to 100 nm from the SEM measurement. Supposing that the CA and CAD molecules are closely packed along the LDH layers, maximum 20,000 molecules (calculation below) can be accommodated in one LDH layer.

Area of an LDH layer: 50 nm × 50 nm × π ~ 7,850 nm^2

Demanded area of a CA or CAD molecule: 0.35 nm × 0.35 nm × π ~ 0.385 nm^2

7,850/0.385 ~ 20,000 unit of CA(D) along a layer of LDH

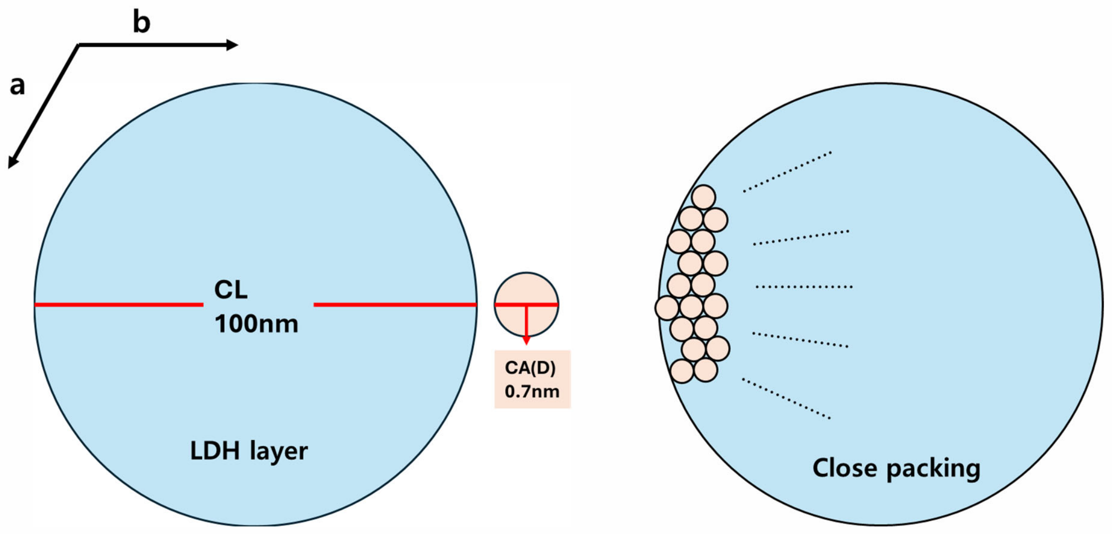

Figure. S2. Schematic diagram to calculate approximate number of CA or CAD molecules between LDH layers. It was hypothesized that one CA or CAD molecule demands cross-sectional area with diameter 0.7 nm based on the molecular dimension and π-π interaction distance obtained from Monte Carlo simulation. The lateral dimension of LDH was approximated to 100 nm from SEM measurement.
